# A 10-Year Prospective Evaluation of Balloon Tube Tamponade and Emergency Injection Sclerotherapy for Actively Bleeding Oesophageal Varices

**DOI:** 10.1155/1989/98263

**Published:** 1989

**Authors:** D. Kahn, P. C. Bornman, J. Terblanche

**Affiliations:** 1 Department of Surgery and the Medical Research Council Liver Research Centre the University of Cape Town and Groote Schuur Hospital Cape Town South Africa; 2 Department of Surgery University of Cape Town Medical School Observatory 7925 South Africa

## Abstract

During a 10 year study period 234 patients were admitted on 371 occasions with a total of 566 acute variceal bleeding episodes. Of these, 173 patients had 343 variceal bleeds which required balloon tamponade to achieve initial control of bleeding during 229 admissions and were then referred for emergency injection sclerotherapy. Sixty-eight percent of these patients had alcoholic cirrhosis and 42% were poor risk Grade C patients. Injection sclerotherapy was performed initially using the rigid Negus oesophagoscope under general anaesthesia and later using the fibreoptic endoscope under light sedation. Definitive control of variceal bleeding was achieved with sclerotherapy during 197 hospital admissions (92%). Of the 17 failures of emergency sclerotherapy, 4 patients died from uncontrolled bleeding and 13 patients underwent major surgical intervention. Definitive control of variceal bleeding was achieved with a single injection treatment in 138 hospital admissions (70%). Complications were mostly of a minor nature and occurred at a rate of 6% per injection treatment. The overall hospital admission mortality was 36%. The majority of patients died due to liver failure. The mortality in patients who required 4 injection treatments to control variceal bleeding was 71%. Injection sclerotherapy is proposed as the emergency treatment of choice for patients whose variceal bleeding continues or recurs after initial conservative management. Patients whose variceal bleeding is not controlled by 2 injection treatments require more major emergency surgery.


*HPB Surgery* 1989, Vol. 1, pp. 207–219
Reprints available directly from the publisher
Photocopying permitted by license only

© 1989 Harwood Academic Publishers GmbH
Printed in Great Britain

### A 10-YEAR PROSPECTIVE EVALUATION OF BALLOON TUBE TAMPONADE AND EMERGENCY INJECTION SCLEROTHERAPY FOR ACTIVELY BLEEDING OESOPHAGEAL VARICES

D. KAHN, P.C. BORNMAN and J. TERBLANCHE*

*Department of Surgery, and the Medical Research Council Liver Research Centre, the University of Cape Town and Groote Schuur Hospital, Cape Town, South Africa*

*(Received 10 October 1988)*

During a 10 year study period 234 patients were admitted on 371 occasions with a total of 566 acute variceal bleeding episodes. Of these, 173 patients had 343 variceal bleeds which required balloon tamponade to achieve initial control of bleeding during 229 admissions and were then referred for emergency injection sclerotherapy. Sixty-eight percent of these patients had alcoholic cirrhosis and 42% were poor risk Grade C patients. Injection sclerotherapy was performed initially using the rigid Negus oesophagoscope under general anaesthesia and later using the fibreoptic endoscope under light sedation. Definitive control of variceal bleeding was achieved with sclerotherapy during 197 hospital admissions (92%). Of the 17 failures of emergency sclerotherapy, 4 patients died from uncontrolled bleeding and 13 patients underwent major surgical intervention. Definitive control of variceal bleeding was achieved with a single injection treatment in 138 hospital admissions (70%). Complications were mostly of a minor nature and occurred at a rate of 6% per injection treatment. The overall hospital admission mortality was 36%. The majority of patients died due to liver failure. The mortality in patients who required 4 injection treatments to control variceal bleeding was 71%. Injection sclerotherapy is proposed as the emergency treatment of choice for patients whose variceal bleeding continues or recurs after initial conservative management. Patients whose variceal bleeding is not controlled by 2 injection treatments require more major emergency surgery.

KEY WORDS: Sclerotherapy, oesophageal varices.

### INTRODUCTION

Emergency injection sclerotherapy has become firmly established as part of the management of acute variceal haemorrhage, particularly if bleeding does not respond to simple conservative measures[1–3]. Definitive control of variceal haemorrhage with this form of therapy is reported to be in excess of 90%[4–9]. This paper reviews the first 10 years of our prospective study of injection sclerotherapy in the emergency management of the subset of bad-risk patients with endoscopically proven active variceal haemorrhage who required Sengstaken balloon tube tamponade for initial control of bleeding. During the 10 years technical variations of sclerotherapy were evaluated and a new technique introduced after 5 years.

*Correspondence to: Professor John Terblanche, Department of Surgery, University of Cape Town Medical School, Observatory 7925, South Africa.

### PATIENTS AND METHODS

### *Patients*

All 306 patients admitted to the adult wards of the hospital with endoscopically proven oesophageal variceal bleeding during the 10 year period August 1975 to August 1985 were evaluated and documented prospectively on computer pro forma sheets. Of these, 234 patients were referred specifically for emergency management of actively bleeding varices. These latter patients were admitted on 371 occasions with a total of 566 acute variceal bleeding episodes (Figure 1). In 223 of the 566 acute bleeding episodes, bleeding stopped after the initial conservative treatment and further emergency therapy was not required. Patients who had either continued variceal bleeding despite the initial conservative therapy or massive variceal bleeding, were treated with immediate tamponade with a Sengstaken-Blakemore balloon tube and were later subjected to emergency injection sclerotherapy. Only this latter group of 173 patients with continued active variceal bleeding on their first or subsequent admission have been included in the present study which evaluated emergency treatment of continued variceal bleeding.

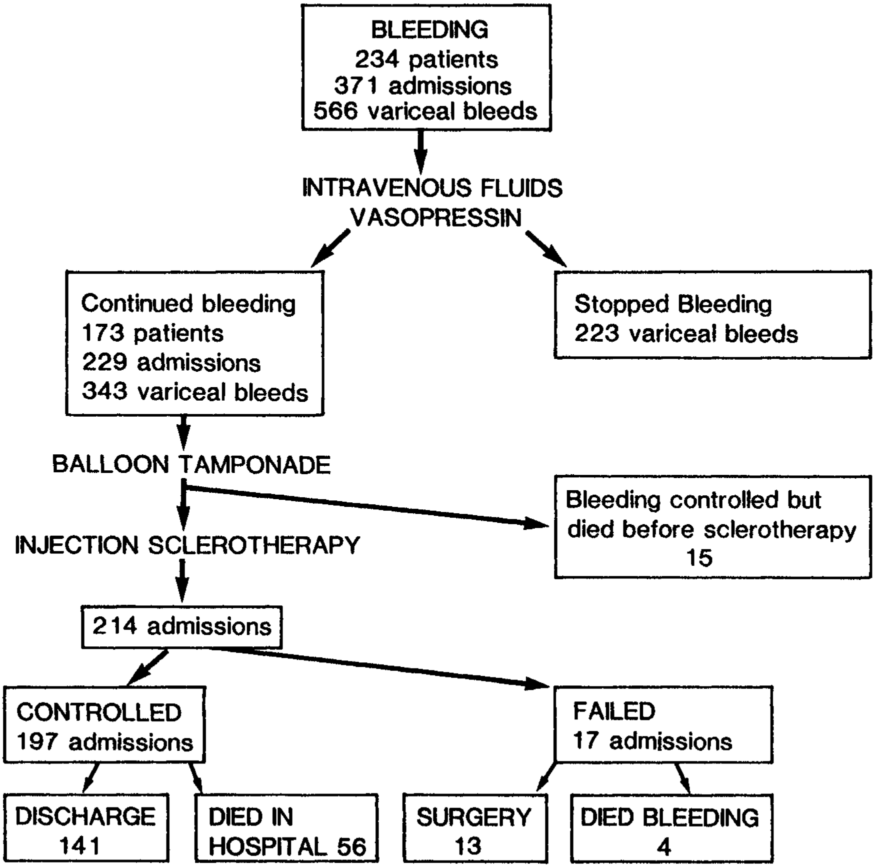

**Figure 1** Treatment and outcome of patients with actively bleeding oesophageal varices.

The subgroups of patients included in the controlled trials all gave informed consent and the trials were approved by the Faculty of Medicine Ethics and Research Committee.

The 173 patients included in this evaluation of emergency sclerotherapy had 343 acute variceal bleeds which were treated with balloon tamponade in 229 admissions during the 10 year analysis period (Figure 1). There were 125 males and 48 females and the mean age was 47.8 ± 1.26 years (range 13–80). A modified Pugh-Child's risk Grade was used to assess the severity of the liver disease[10]. On admission 34 patients were Grade A, 60 were Grade B and 67 were Grade C. The risk grade could not be determined in 12 patients. The aetiology of the portal hypertension is shown in Table I. Histological evidence of cirrhosis was found in 139 patients, and of these 116 patients had a definite history of alcohol abuse. A diagnosis of extrahepatic portal venous obstruction was based on normal liver histology and/or angiographic findings.

### *Initial management of the acute variceal bleeding episode*

Patients with suspected acute oesophageal variceal bleeding were admitted, whenever possible, to an intensive care or high care unit. Initial management included resuscitation with crystalloids and blood, and medication to combat encephalopathy. Most patients received intravenous vasopressin prior to emergency endoscopy. During the first two years bolus doses of vasopressin were used[6], but since then it has been infused continuously intravenously (0.4 units per minute)[9]. Patients who did not have varices and those with varices but who were bleeding from other lesions were excluded from the study. In patients with varices that had stopped bleeding, the vasopressin infusion was continued for 24–48 hours. A Sengstaken-Blakemore balloon tube was only inserted in those patients with active variceal bleeding at the time of emergency endoscopy. This latter group of patients underwent emergency injection sclerotherapy between 6 and 24 hours after inserting the Sengstaken tube. These 173 patients constitute the present study groups (Figure 1). Resuscitation was continued and liver failure treated with Neomycin and/or Lactulose as required.

### *Injection Sclerotherapy Techniques*

During the 10-year study period mainly 2 sclerotherapy techniques were used, with either a rigid or a flexible endoscope. The technique of emergency injection sclerotherapy using the rigid Negus oesophagoscope has been described in detail elsewere[6,11]. A standard anaesthetic technique was used[11]. After removing the

**Table 1** Aetiology of the portal hypertension.

| | |
|---|---|
| Alcoholic cirrhosis | 116 |
| Cryptogenic cirrhosis | 23 |
| Portal vein thrombosis | 18 |
| Chronic active hepatitis | 5 |
| Other | 3 |
| Unknown | 8 |
| TOTAL | 173 |

Sengstaken tube, a 50cm rigid Negus oesophagoscope, modified with a slot[12], was passed to the oesophagogastric junction and the sclerosant, ethanolamine oleate, injected directly into the varix. In most cases three variceal channels presented for injection and on any one occasion a total of between 6 and 20 ml of sclerosant was injected per treatment session. The oesophagoscope was left in situ for a further 5 minutes to compress the varices. In the first 2 years of the study a Sengstaken tube was often inserted following sclerotherapy but was avoided thereafter because of the associated dangers[9].

Fibrooptic endoscopic injection sclerotherapy has been used increasingly in recent patients, some of whom were included in a controlled trial comparing the two techniques[13]. Fibreoptic sclerotherapy was used almost exclusively in the latter 5 year period. Fibreoptic sclerotherapy was performed under light sedation using an unmodified endoscope. The sclerosant, ethanolamine oleate, was injected both intra- and paravariceally and a total of between 5 and 20 ml of sclerosant was injected during any one treatment session[14]. In 1984, for four months, during and after a visit by Paquet, a few patients were injected using his paravariceal technique[15] with aethoxysclerol as the sclerosing agent.

### RESULTS

### *Control of Bleeding*

A Sengstaken-Blakemore balloon tube was inserted for initial control of 343 variceal bleeding episodes during 229 admissions. In 15 patients the Sengstaken tube controlled the bleeding but the patients died before undergoing sclerotherapy. Initial balloon tamponade followed by emergency injection sclerotherapy failed to control bleeding in 17 admissions. Definitive control of variceal bleeding was achieved with sclerotherapy during the hospitalisation period in 197 admissions (92%). Of the 17 failures of tamponade and sclerotherapy, 4 patients died from uncontrolled bleeding and 13 patients required emergency surgical intervention in the form of a devascularisation procedure in 10 patients, a portacaval shunt in 2 patients and a distal splenorenal shunt in 1. The variceal bleeding ceased post-operatively in all the cases treated surgically but 8 patients (61%) died from causes other than variceal bleeding. In 2 of the 4 patients who died from uncontrolled bleeding, the bleeding continued with the Sengstaken tube in place. These are the only 2 patients in whom the balloon tube failed to achieve temporary control of variceal bleeding. Fifty-six patients died during the hospitalisation period after definitive control of the variceal bleeding was achieved.

Definitive control of variceal bleeding was achieved with a single injection treatment in 70% of hospital admissions (138 admissions). Two injection treatments were required for definitive control on 40 occasions and 3 or 4 injection treatments on 16 and 4 occasions respectively.

### *Complications*

A detailed evaluation of our overall experience of the complications of sclerotherapy is presented elsewhere[16]. These complications were mostly of a minor nature. During the 10-year period the 173 patients under evaluation for acute variceal bleeding

**Table 2** Outcome after initial balloon tamponade control of bleeding.

| | *Hospital Admissions* |
|---|---|
| Definitive control of bleeding | 197 |
| Died before sclerotherapy | 15 |
| Failed sclerotherapy: | |
| Died from uncontrolled bleeding | 4 |
| Required surgical intervention | 13 |
| TOTAL | 229 |

received a total of 311 emergency injection sclerotherapy treatments. There were 19 major oesophageal complications; the morbidity per injection was therefore 6.1%. No frank oesophageal perforations were associated with emergency sclerotherapy.

An injection site leak, as described previously[9], represents a localised, perioesophageal leak and is demonstrated on barium or gastrografin swallon. It occurred after emergency injection sclerotherapy in 14 patients. This complication was suspected in patients with persistant pain and pyrexia after sclerotherapy and the diagnosis was confirmed on gastrografin swallow. All patients settled on conservative treatment which included nil by mouth, intravenous fluids, hyperalimentation (when indicated) and antibiotics. Subsequent sclerotherapy was delayed and there was an associated mortality[16].

Mild oesophageal stenosis demonstrated by barium swallow occurred in 5 patients. These patients complained of dysphagia following sclerotherapy. In all the patients it was a transient phenomenon and none of the patients required dilatation.

Superficial mucosal slough was noted on subsequent endoscopy in a number of patients, and at times resulted in repeated long-term sclerotherapy being delayed. One patient continued to bleed from the mucosal slough and required a devascularisation procedure; he eventually died from multiorgan failure.

*Mortality (Tables 3 and 4)*

Eighty-three of the 173 patients who required balloon tamponade for initial control of variceal haemorrhage died during the 229 hospital admissions. Overall hospital admission mortality in this difficult to treat subset of patients with continued active variceal bleeding was therefore 36%. However 15 patients died while temporarily controlled with the Sengstaken-Blakemore tube in situ before receiving sclerotherapy. Thus the hospital admission mortality after acute injection sclerotherapy was 32%. Eight patients died after various surgical procedures from causes other than variceal bleeding. Only 4 patients (2.3%) died from uncontrolled variceal bleeding. Thirty-eight of the hospital deaths after sclerotherapy were modified Pugh-Child's Grade C at the initial assessment, 27 were Grade B and 3 were Grade A. The latter 3 patients, although Grade A at their initial assessment had all deteriorated to modified Pugh-Child's Grade B during the admission for the massive variceal bleed which preceeded their death.

Patients who only required a single injection sclerotherapy treatment had a mortality of 22% whereas the mortality was 71 when 4 injections were needed (Table 3).

**Table 3** Mortality related to number of injection sclerotherapy treatments undertaken.

| | *Number of Admissions* | *Deaths* | *Mortality* |
|---|---|---|---|
| 1 Injection Treatment | 143 | 31 | 22% |
| 2 Injection Treatments | 48 | 25 | 52% |
| 3 Injection Treatments | 16 | 7 | 44% |
| 4 Injection Treatments | 7 | 5 | 71% |
| TOTAL | 214 | 68 | |

The causes of death are shown in Table 4. The majority of patients died of liver failure. In 32 patients the liver failure was associated with multiorgan failure. Only 4 patients (2.3%) died as a result of uncontrolled bleeding.

**Table 4** Causes of death.

| | *Patients* |
|---|---|
| Liver Failure: | |
| Primary | 33 |
| Associated Multiorgan Failure | 32 |
| Cardiorespiratory | 12 |
| Bleeding | 4 |
| Sepsis | 2 |
| TOTAL | 83 |

### DISCUSSION

The use of injection sclerotherapy for oesophageal varices was first described by Crafford and Frenckner in 1939[17]. Their report of the elective treatment of a single patient who had previously bled from oesophageal varices, stimulated considerable interest. However isolated favourable reports were overshadowed by the advent and widespread adoption of portacaval shunting at that time. In 1973 Johnston and Rodgers[4] reported excellent results with injection sclerotherapy for acute variceal bleeding. This was followed in 1979 by the inital report on emergency sclerotherapy from our group[6] and thereafter interest in sclerotherapy as an alternative to surgery was revived. Since then injection sclerotherapy has become firmly established as the treatment of choice in the emergency management of bleeding oesophageal varices. Three controlled trials have shown injection sclerotherapy to be superior to conventional treatment, including balloon tamponade, in the emergency management of acute variceal bleeding[1–3]. Alternative therapeutic modalities in the management of bleeding oesophageal varices, including percutaneous transhepatic obliteration[18], emergency portosystemic shunting[19–21] and devascularisation and transection procedures[22–24], have been associated with a signficant morbidity and mortality and, other than with shunting, a high rate of rebleeding. A recent controlled trial has rekindled interest in portacaval shunting in poor risk patients[25].

This paper reports a 10-year prospective evaluation of emergency injection sclerotherapy in patients with acute bleeding oesophageal varices who required a Sengstaken tube for initial control of haemorrhage. Most of the survivors went on to have repeated sclerotherapy as part of their long-term management, which will be our hospital in August 1975 because of the disappointing high morbidity and mortality reported with conservative measures, including our own experience of the use of the Sengstaken tube alone[26], and both our experience and the reported experience with operative procedures, such as direct operations on the varices[22,23] and emergency portacaval shunting[19].

In the present study of emergency management, definitive control of variceal bleeding was achieved in 92% of hospital admissions where balloon tube tamponade was followed by emergency sclerotherapy and usually only a single injection treatment was required (70%). Johnston and Rodgers[4], Paquet and Oberhammer[5], Terblanche et al[6,9], and Spence et al[27] have also reported control rates of over 90 percent. The present results, with more patients, confirm our earlier publications[6,9] showing the superiority of combined sclerotherapy and balloon tamponade when compared with the use of the Sengstaken tube alone, where the rate of rebleeding after removal of the tube was 60% in our experience [26].

The total hospital admission mortality rate for sclerotherapy treated patients was 32%, which compares favourably with the mortality rate of 60% in patients previously treated with a Sengstaken tube only[26]. The mortality rates in other studies of sclerotherapy have varied between 14 and 28%[4–6,9,27], and depended on the status of the liver function of the patients included. These mortality rates compare favourably with that achieved after any operative procedure in these very ill patients.

The majority of our patients were alcoholic cirrhotics with severely compromised liver function and liver failure was the major cause of death. Only 4 patients died from uncontrolled bleeding. It is important to note that in many patients the liver failure was preceeded by a variceal bleed and thus the actual categorisation of the cause of death remains difficult.

In this study patients who received four injection treatments had an unacceptably high mortality. In an earlier study we reported a mortality rate of 69% in patients who received three or four injection treatments with a mortality of nearly 90% if good risk modified Pugh-Child's A Grade patients are excluded[28]. The present findings support our previous recommendation that those patients who continue to bleed from varices after two injection treatments should have a balloon tube reinserted and be considered for other treatment modalities. The surgical options recommended in these high risk patients are a transection procedure[23,24] or a portacaval shunt[20,21,25,29].

Which injection sclerotherapy technique is best, remains unresolved. In the present study varices were injected either with the modified rigid Negus oesophagoscope under general anaesthetic and the sclerosant injected intravascularly into the varix, or with the flexible endoscope under light sedation and the sclerosant injected using a combined intra- and paravariceal injection technique[14]. These two techniques were compared in a group of 70 patients in a randomised controlled trial and were found to be equally effective in controlling acute variceal bleeding and in the long term eradication of oesoophageal varices[13]. Although the modified rigid oesophagoscope may be more efficient in controlling recurrent active acute bleeding from varices in some difficult patients, because of the more controlled situation under anaesthesia with endotracheal intubation, we would only recommend the use of a rigid endoscope if considerable expertise is available. Where a flexible endoscope technique is used, we currently protect the airway with an endotracheal tube in all stuperose or comatose patients. A paravariceal technique with the flexible oesophagoscope has also been shown by others to be highly effective[2,5,15]. Further controlled trials are required to determine the best technique and the best sclerosant.

Since the present 10 year evaluation closed we have been evaluating sclerotherapy treatment at the time of the first diagnostic endoscopy, whenever practical. Although this approach appears logical there is currently no controlled trial data to support its use. For the failures of sclerotherapy we advocate simple staple gun transection of the oesophagus, but portacaval shunting requires re-evaluation in the light of the satisfactory results reported by Cello et al in their trial comparing it with sclerotherapy in poor risk patients[25], and the data from two other recent Californian studies[21,29].

In conclusion, we believe that emergency injection sclerotherapy is the treatment of choice for patients in whom variceal bleeding continues or recurs following initial conservative treatment and who require balloon tamponade for initial control of bleeding. The role of immediate sclerotherapy at the time of the first diagnostic endoscopy remains to be established. The failures, defined as those patients not controlled by 1 or 2 injection sclerotherapy treatments, should be subjected to more major surgery. Here the choice between a shunt and a transection operation also remains to be defined by controlled trials.

### *Acknowledgements*

The study was supported by the South African Medical Research Council and the Staff Research Fund of the University of Cape Town. We are grateful to our many colleagues who have assisted us with the study over the years.

### *References*

1. Barsoum, M.S., Bolous, F.I., El-Rooby, A.A., Rizk-Allah, M.A. and Ibrahim, A.S. (1982) Tamponade and injection sclerotherapy in the management of bleeding oesophageal varices. *British Journal of Surgery,* **69**, 76–78
2. Paquet, K-J. and Feussner, H. (1985) Endoscopic sclerosis and esophageal balloon tamponade in acute hemorrhage from esophagogastric varices: A prospective controlled randomised trial. *Hepatology,* **5**, 580–583
3. Larson, A.W., Cohen, H., Zweiban, B., Chapman, D., Gourdji, M., Korula, J. and Weiner, J. (1986) Acute esophageal variceal sclerotherapy. Results of a prospective randomised controlled trial. *Journal of the American Medical Association,* **255**, 497–500
4. Johnston, G.W. and Rodgers, H.W. (1973) A review of 15 years' experience in the use of sclerotherapy in the control of acute haemorrhage from oesophageal varices. *British Journal of Surgery,* **60**, 797–800
5. Paquet, K-J. and Oberhammer, E. (1978) Sclerotherapy of bleeding oesophageal varices by means of endoscopy. *Endoscopy,* **10**, 7–12
6. Terblanche, J., Northover, J.M.A., Bornman, P.C., Kahn, D., Barbezat, G.O., Sellars, S.L. and Saunders, S.J. (1979) A prospective evaluation of injection sclerotherapy in the treatment of acute bleeding from esophageal varices. *Surgery,* **85**, 239–245
7. Johnson, A.G. (1979) Injection sclerotherapy in the emergency and elective treatment of oesophageal varices. *Annals of Royal College of Surgeons of England,* **59**, 497–501
8. Clark, A.W., Macdougall, B.R.D., Westaby, D., Michell, K.J., Silk, D.B.A., Strunin, L., Dawson, J.L. and Williams, R. (1980) Prospective controlled trial of injection sclerotherapy in patients with cirrhosis and recent variceal haemorrhage. *Lancet,* ii, 552–554
9. Terblanche, J., Yakoob, H.I., Bornman, P.C., Stiegmann, G.V., Bane, R., Jonker, M.A.T., Wright, J. and Kirsch, R.E. (1981) Acute bleeding varices: A five year prospective evaluation of tamponade and sclerotherapy. *Annals of Surgery,* **194**, 521–530

10. Terblanche, J., Northover, J.M.A., Bornman, P.C., Kahn, D., Silber, W., Barbezat, G.O., Sellars, S.L., Campbell, J.A.H. and Saunders, S.J. (1979) A prospective controlled trial of sclerotherapy in the long-term management of patients after variceal bleeding. *Surgery Gynaecology and Obstetrics*, **148**, 323–333
11. Terblanche, J. (1981) Treatment of esophageal varices by injection sclerotherapy. In: Maclean, LD, ed. Advances in Surgery, Vol 15, Chicago: Year Book Publishers, pp 257–291
12. Bailey, M.E. and Dawson, J.L. (1975) Modified oesophagoscope for injecting oesophageal varices. *British Medical Journal*, **2**, 540–542
13. Bornman, P.C., Kahn, D., Terblanche, J., Worthley, C., Spence, R.A. and Krige, J.E.J. (1988) Rigid versus fiberoptic endoscopic injection sclerotherapy. A prospective randomized controlled trial in patients with bleeding esophageal varices. *Annals of Surgery*, **208**, 175–178
14. Terblanche, J., Bornman, P.C., Jonker, M.A.T., Kirsch, R.E. and Saunders, S.J. (1982) Injection sclerotherapy of esophageal varices. *Seminars in Liver Disease*, **2**, 233–241
15. Paquet, K-J. (1983) Endoscopic paravariceal injection sclerotherapy of the esophagus – indications, technique, complications: results of a period of 14 years. *Gastrointestinal Endoscopy*, **29**, 310–315
16. Kahn, D., Jones, B., Bornman, P.C. and Terblanche, J. (1989) Incidence and management of complications after injection sclerotherapy: A 10 year prospective evaluation. *Surgery*, **104**, In Press
17. Crafford, C. and Frenckner, P. (1939) New surgical treatment of varicose veins of the oesophagus. *Acta Otolaryngologica*, **27**, 422–424
18. Smith-Laing, G., Scott, J., Long, R.G., Dick, R. and Sherlock, S. (1981) Role of percutaneous transhepatic obliteration of varices in the management of hemorrhage from gastroesophageal varices. *Gastroenterology*, **80**, 1031–1036
19. Giles, G.R., Brennan, T.G. and Losowsky, M.S. (1973) Interposition teflon mesenteric caval shunt for bleeding oesophageal varices. *British Journal of Surgery*, **60**, 649–652
20. Orloff, M.J., Charters, A.C., Chandler, J.G., Condon, J.K., Grambort, D.E., Modafferi, T.R., Levin, S.E., Brown, N.B., Sviokla, S.C. and Knox, D.G. (1975) Portacaval shunt as emergency procedure in unselected patients with alcoholic cirrhosis. *Surgery Gynecology and Obstetrics*, **141**, 59–68
21. Orloff, M.J. and Bell, R.H. (1986) Long-term survival after emergency portacaval shunting for bleeding varices in patients with alcoholic cirrhosis. *American Journal of Surgery*, **151**, 176–183
22. George, P., Brown, C., Ridgeway, G., Crofts, B. and Sherlock, S. (1973) Emergency oesophageal transection in uncontrolled variceal haemorrhage. *British Journal of Surgery*, **60**, 635–640
23. Pugh, R.N.H., Murray-Lyon, I.M., Dawson, J.L., Pietroni, M.C. and Williams, R. (1973) Transection of the oesophagus for bleeding oesophageal varices. *British Journal of Surgery*, **60**, 646–649
24. Johnston, G.W. (1982) Six years' experience of oesophageal transection for oesophageal varices, using a circular stapling gun. *Gut*, **23**, 770–773
25. Cello, J.P., Grendell, J.H., Crass, R.A., Weber, T.E. and Trunkey, D.D. (1987) Endoscopic sclerotherapy versus portacaval shunt in patients with severe cirrhosis and acute variceal hemorrhage. Long-term follow-up. *New England Journal of Medicine*, **316**, 11–15
26. Novis, B.H., Duys, P., Barbezat, G.O., Clain, J., Bank, S. and Terblanche, J. (1976) Fibreoptic endoscopy and the use of the Sengstaken tube in acute gastrointestinal haemorrhage in patients with portal hypertension and varices. *Gut*, **17**, 258–262
27. Spence, R.A.J., Anderson, J.R. and Johnston, G.W. (1985) Twenty-five years of injection sclerotherapy for bleeding varices. *British Journal of Surgery*, **72**, 195–198
28. Bornman, P.C., Terblanche, J., Kahn, D., Jonker, M.A.T. and Kirsch, R.E. (1986) Limitations of multiple injection sclerotherapy sessions for acute variceal bleeding. *South African Medical Journal*, **70**, 31–36
29. Sarfeh, I.J., Rypins, E.B. and Mason, G.R. (1986) A systematic appraisal of portacaval H-graft diameters. Clinical and hemodynamic perspectives. *Annals of Surgery*, **204**, 356–363

### INVITED COMMENTARY

This is an important ten year audit of the emergency management of bleeding varices from a centre which did much to establish the worth of this technique. These excellent results support my long held belief that injection sclerotherapy is the

"best buy" for the acute bleeding episode. A 92 per cent bleeding control rate in this high risk subgroup of patients who fail to stop bleeding without tamponade equals or betters that obtained in most centres for 'all comers" with variceal bleeding. Although only four patients are recorded as having died of uncontrolled bleeding, there were other deaths recorded as liver failure where further bleeding preceded organ failure. In this situation where bleeding precedes rather than succeeds liver failure, perhaps these deaths should also be attributed to bleeding, although this is a difficult area of definition. We should not lose sight of the fact that oesophageal tamponade controlled bleeding initially in 99 per cent of patients; this modality still has an important holding role to play. The tendency to do immediate sclerotherapy without tamponade is gaining support but it is not always practical or possible if one believes in early referral of these patients to centres with a special interest in portal hypertension. The evolution of management in this series over a ten year period is similar to that of many centres – the abandonment of post-injection tamponade, the decreasing use of the rigid instrument, the flirtation with sclerosing agents other than ethanolamine oleate, etc. The problem of how best to manage the patients who continue bleeding in spite of sclerotherapy remains. Of the 17 patients who continued to bleed after acute injection sclerotherapy, 13 were subjected to surgery and five survived. The authors give the impression that perhaps better results could have been obtained by a more aggressive approach, that is, "Patients not controlled by one or two injection sclerotherapy treatments should be subjected to more major surgery, either shunt or transection." However surgery can only help in stopping bleeding, and in this series only four patients died from bleeding. The majority, 70 per cent of the whole series and 61 per cent of those treated surgically, died of liver failure where operation has nothing to offer. Indeed the 61 per cent mortality in the surgical group does not differ greatly from the 71 per cent mortality even after four injection. The problem is that persistent bleeding often indicates a severe degree of liver dysfunction and in addition is sometimes the result of post-sclerotherapy oesophageal ulceration, neither situation being condusive to operation. In addition it is commonly Child's Grade C patients who present this problem of recurrent bleeding and unfortunately few Grade C patients survive emergency surgery. Oesophageal transection of a recently injected oedematous and possibly ulcerated oesophagus is hazardous and even shunt surgery does not necessarily stop bleeding from an oesophageal ulcer overlying a varix. Perhaps there is a place for resorting to re-insertion of the Sengstaken tube for a few days with intermittent tamponade in these difficult rebleeders. The number of patients failing with sclerotherapy is so small that it will be difficult to get a scientific answer to as the best therapy in this situation.

George W. Johnston
Royal Victoria Hospital
Grosvenor Road
Belfast
BT12 6BA

### INVITED COMMENTARY

The data presented by Kahn and colleagues summarized a 10 year experience of sclerotherapy in management of acute variceal bleeding. Since the group at Capetown first published their work on sclerotherapy in 1979, accumulation of data from around the world has clearly established the efficacy of the procedure. What is urgently needed, however, are criteria for determination of failure of sclerotherapy, without which its true role in the overall management of variceal bleeding remains undefined. Unlike surgical treatment where the effects are immediate, obliteration of varices with sclerotherapy takes multiple sessions over time. Should recurrent bleeding from residual patent varices be called failure of sclerotherapy or inadequate sclerotherapy?

It seems to this author that there are two possible situations where one may consider sclerotherapy to be a failure. The first is more clear cut: failure to stop bleeding with sclerotherapy during an acute episode; and the second is recurrent hemorrhage during a course of chronic sclerotherapy.

In the series of Kahn et al a single injection sufficed to control 70% of the bleeding episodes, while in 22% multiple injections were required. In 8% sclerotherapy simply failed to control bleeding, and in 2.3% the patients died from continued bleeding. The experience elsewhere, as well as our own, supports the approximation that 5–10% of all patients bleeding from varices cannot be controlled acutely by sclerotherapy. The important question, therefore, is why doesn't sclerotherapy work in this group of patients?

In the Syracuse series [1] of 53 consecutive patients admitted with bleeding from esophageal varices and related lesions, five required emergency surgery due to failure to stop bleeding with sclerotherapy. The factors common to this small group are: massive ascites (6.3 ± 1.5 L), massive blood loss before control (9 ± 3 Units), a portal pressure of 27.6 ± 6.7 cm $H_2O$, and poor liver function (one patient in B and four patients in C, Pugh-Child's classification). Conditions such as gastric variceal bleeding and portal hypertensive gastropathy are unsuitable for sclerotherapy and are not included in the Kahn series.

In our investigation of the sources of recurrent hemorrhage during chronic sclerotherapy [1], we found that bleeding most often came from persistently patent varices, and that eradication of these varices was ultimately achievable in 15 out of 18 patients with continued sclerotherapy. Recurrent bleeding was rare after eradication. New varices, defined as vessels not seen in previous videorecordings, occurred in 2 patients and were never the source of bleeding. We feel that recurrent bleeding from patent varices should not be branded as failiure of sclerotherapy, particularly in view of the penalty that may be associated with changing to a more invasive treatment modality. In our view, true failures would include bleeding from deep ulcerations caused by sclerotherapy and from sources other than esophageal varices for which sclerotherapy is ineffective.

Kahn and colleagues suggested that surgical treatment should be considered when sclerotherapy has failed to control acute bleeding. Would such patients fare betters with surgical treatment? Such an approach would seem reasonable but unfortunately no data were presented to answer this question. In the Syracuse series, surgical treatment of patients who continued to bleed after maximal sclerotherapy carries a mortality of 60%, and at a high cost. It may well be that these patients do not respond well to any form of treatment, irrespective of the degree of invasiveness, but controlled investigation into options other than surgery, such as combined radiological, pharmacological and surgical treatment[2], would be necessary to improve the outcome for this difficult group of patients.

Raphael S. Chung, MD, FACS
Department of General Surgery
Cleveland Clinic Foundation
Cleveland, OH 44195-5043

*References*

1. Chung, R.S. and Dearlove, J. (1988) The sources of recurrent hemorrhage during chronic sclerotherapy. *Surgery* (October issue).
2. Berman, H.L., DelGuercio, R.M. and Katz, S.G. et al. (1988) Minimally invasive devascularization for variceal bleeding that could not be controlled with sclerotherapy. *Surgery,* **104**, 500–506

### INVITED COMMENTARY

Bleeding oesophageal varices still present a challenge to surgeons and gastroenterologists and it is good to see that the good initial results of balloon tamponade and sclerotherapy have been maintained in Capetown, over the last 10 years in a large number of patients. In most people's experience, about one third of patients have stopped bleeding when they reach hospital, one third are bleeding slightly and one third bleeding heavily. Ideally the varices should be injected at the same time as the diagnostic endoscopy and it is interesting that the authors mention that they are looking at that area. However, there are still patients in whom the bleeding is heavy and the endoscopic view is not good enough to allow a safe and effective injection. The 4 lumen modification of the Sengstaken Blakemore tube is a safe and effective means of control in experienced hands and it should only be removed just before sclerotherapy.

The patients in this South African study belong to this difficult group who required balloon tamponade because they failed to stop bleeding, either spontaneously or after vasopressin. However, many clinicians do not now consider vasopressin alone, to be satisfactory and have stopped using it. Metoclopramide[1] and the more expensive drug, somatostatin, look promising alternatives.

This paper highlights other key problems in the management of bleeding varices. First, despite a 92% control of bleeding by sclerotherapy the hospital mortality is still 36% and these patients mainly die of liver failure. The speed of referral to hospital and the amount of blood lost before the patient reaches expert care may be a factor: the geography and an efficient referral and transfer system may partly account for the low in-hospital mortality reported by Johnston from Belfast. The other problem is those patients who continue to bleed and need repeated injection sessions or have to go to emergency surgery. Kahn documents what many have suspected, that the mortality increases if more than one injection session is needed to control bleeding, rising to 71% after 4 sessions. At the same time, those few patients (8%) who then need a transection/devascularisation operation, because bleeding is uncontrolled by sclerotherapy, also have a high mortality because of their general condition and because oesophageal oedema or ulceration makes transection difficult or dangerous. Preventing a rebleed in hospital is as important in reducing the mortality from bleeding varices as it is from bleeding peptic ulcer. The challenge is to identify these patients early on and, perhaps, take them straight to surgery, but so far no criteria have been found. However, in our experience, a number have developed an extra complication of their cirrhosis such as hepatoma or portal vein thrombosis, and these conditions are also difficult to diagnose in an emergency.

The best technique of injection is undecided and the Cape Town trial showed equal results with the rigid endoscope and general anaesthesia or flexible instrument under sedation. However, occasionally the rigid instrument is more effective in patients with large varices or those which bleed as soon as the tamponade balloon is deflated. In patients who are very confused and violent, a general anaesthetic is safer and makes effective injection easier.

Kahn and colleagues have shown the effectiveness of the tamponade/sclerotherapy regime; but the answer to preventing and treating bleeding varices in the future is likely to be pharmacological. The present trend is towards immediate injection, as soon as bleeding has been controlled by drugs, while balloon tamponade is reserved for those few who do not repond. Failure of the first injection or rebleeding while in hospital should be treated quickly and effectively, by surgery if necessary.

Alan G Johnson
University Surgical Unit
The University of Sheffield
Royal Hallamshire Hospital
Sheffield S1D 2JF

### *References*

1. Hosking, S.W., Doss, W., El-Zeiny, H., Robinson, P., Barsoum, M.S. and Johnson, A.G. (1988) Pharmacological constriction of the lower oesophageal sphincter: a simple method of arresting variceal haemorrhage. *Gut,* **29**, 1098–1102

*Accepted by S. Bengmark 12 October 1988.*

